# Cardio-Thyrotoxicosis Syndrome: A Review of Thyrotoxic Cardiovascular Disease

**DOI:** 10.7759/cureus.37659

**Published:** 2023-04-16

**Authors:** Pradnya Brijmohan Bhattad, Mazen Roumia

**Affiliations:** 1 Cardiovascular Medicine, Saint Vincent Hospital, UMass Chan Medical School, Worcester, USA

**Keywords:** atrial fibrillation, thyrotoxic cardiovascular disease, tachycardiomyopathy, thyrotoxicosis, heart failure

## Abstract

Thyrotoxicosis, an endocrine disorder characterized by elevated serum thyroid hormone levels of tri-iodothyronine (T3) and/or thyroxine (T4), can impact cardiovascular health in several ways. The cardiovascular system is often severely targeted by the thyrotoxic state, and the term “Cardio-thyrotoxic syndrome” has been proposed to encompass the various cardiovascular disease states resulting from thyrotoxicosis. In this review, we discuss various cardiovascular disorders resulting from the effects of thyrotoxicosis. It is important to keep a high index of suspicion for thyroid disorder in the setting of new atrial fibrillation, heart failure, and tachycardia-induced cardiomyopathy. Management of cardio-thyrotoxicosis involves control of heart rate and blood pressure and treatment of acute cardiovascular complications. Thyroid-specific therapy to achieve a euthyroid state will not only improve but even potentially reverse cardiovascular abnormalities.

## Introduction and background

Thyrotoxicosis is a hypermetabolic state characterized by elevated T3 and/or T4 serum levels due to either an endogenous or an exogenous etiology. Hyperthyroidism due to thyroid gland overactivity resulting in endogenous overproduction of thyroid hormones is a leading cause of thyrotoxicosis. However, thyrotoxicosis and hyperthyroidism are not synonymous because thyrotoxicosis also includes exogenous causes of increased serum T3 and T4 levels in the absence of thyroid glandular overactivity [1]. Thus, all hyperthyroid cases can be considered thyrotoxic, but all thyrotoxicosis cases may not be attributed to hyperthyroidism. Causes of thyrotoxicosis not attributable to hyperthyroidism include trophoblastic tumors, exposure to iodine, thyroiditis, and drug side effects [2]. Causes of hyperthyroidism include Grave’s disease, toxic adenoma, and toxic multinodular goiter [2,3]. Thyroid storm is a severe form of thyrotoxicosis that presents with altered sensorium and carries a high degree of morbidity and mortality with delayed treatment [4].

## Review

Thyrotoxic cardiovascular disease

The heart is one of the most important target organs affected by the thyrotoxic state [5]. Thyrotoxicosis may lead to sinus tachycardia, accelerated hypertension, cardiomyopathy (often tachycardiomyopathy), atrial fibrillation, thromboembolism, conduction disturbances manifesting as electrocardiographic changes such as atrioventricular nodal block, and heart failure [6,7]. Thyrotoxicosis results in increased systolic blood pressure, decreased systemic vascular resistance, diastolic blood pressure (thus increased pulse pressure), and increases in left ventricular contractility, blood volume, myocardial oxygen consumption, and cardiac output [8-10].

Thyroid excess and cardiovascular issues

Thyrotoxicosis, when associated with cardiac conditions, is also termed ‘Cardio-thyrotoxicosis’ by some authors [8,11]. Thyrotoxicosis is very well associated with hypercoagulable states with an elevation of coagulation factors leading to rare and transient intracardiac thrombi [6]. Small intracardiac thrombi may resolve completely on antithyroid medications, but larger thrombi also need anticoagulant therapy [6,12,13]. Cardiac conduction issues may be reversed with anti-thyroid drug therapy, radioiodine (I-131), and subtotal thyroidectomy in many cases [12]. Table 1 shows the clinical cardiovascular manifestations of thyrotoxicosis. 

**Table 1:** Clinical cardiovascular features of thyrotoxicosis [Ref: 7,9,10]

Clinical cardiovascular features of thyrotoxicosis:
Palpitations
Hyperdynamic precordium
Intolerance to exercise with dyspnea on exertion
Elevated systolic blood pressure
Ventricular hypertrophy
Atrial fibrillation
Lower extremity edema
Angina
Congestive heart failure

Mechanism of action of thyroid hormones

Effects of thyroid hormones are primarily mediated by the binding of T3 to specific nuclear thyroid hormone receptors. T4 itself is inactive but acts as a prohormone for the generation of the active hormone T3. Possible non-receptor-mediated effects of thyroid hormone have been proposed but are not elucidated. Thyroid hormone receptors act as hormone-responsive transcription factors. Once T3 binds to its receptor, the hormone-receptor complex interacts with specific deoxyribonucleic acid (DNA) sequences to modulate gene expression, which may either inhibit or stimulate the transcription of specific genes [5,14-16]. The effects of thyroid hormones are synergistic with those of the sympathetic nervous system. Beta-blockers are widely used for the treatment of many symptoms of hyperthyroidism.

Atrial fibrillation and thyrotoxicosis

Various studies show a strong binding relationship between thyrotoxicosis and atrial fibrillation. Atrial fibrillation is a clinical finding in many patients with thyroid storm, and when combined with sinus tachycardia, it can lead to cardiomyopathy presenting with low cardiac output [8,17,18]. Accelerated atrial repolarization and potassium depletion are the probable mechanisms leading to a high occurrence of atrial disorders like atrial fibrillation and atrial flutter in patients with thyrotoxicosis [19]. Studies have shown that thyrotoxicosis and thyroid storm lead to atrial fibrillation and subsequent cardiac arrest, and cardiac insufficiency [20,21]. In subclinical hyperthyroidism in the elderly, the 10-year risk of atrial fibrillation is three-fold when compared to those with normal TSH levels [17,22-23]. So, in patients with lone atrial fibrillation, a thyroid screening to rule out thyrotoxicosis is prudent [7]. Also, if patients do not revert to normal sinus rhythm on their own, elective cardioversion should be tried after bringing the patient back into a euthyroid state [22-24].

Thyroid excess and heart block

Patients may present with PR interval prolongation on a random electrocardiogram (ECG), or either a Type II or Type III block secondary to interstitial inflammation of the atrioventricular (AV) node or the bundle of HIS in hyperthyroidism [22-24]. Abnormalities in AV conduction leading to heart block in such patients may reverse on their own with antithyroid medications without any further need for cardiac pacing [24-25]. Sick sinus syndrome has been reported as a rare complication of thyrotoxicosis, which may be reversible upon achieving a euthyroid state, obviating the need for pacemaker implantation [26].

Thyrotoxicosis, heart failure, and tachycardiomyopathy

In patients with thyroid excess, congestive heart failure and thromboembolism pose a high risk of morbidity and mortality [7]. The functional left ventricular reserve is hampered in the hyperdynamic circulatory state of thyroid excess, and it may be only partially responsive to beta-blockers. Though thyrotoxicosis causes a hyperdynamic circulatory state, there is an occurrence of heart failure secondary to elevated sodium [14-16]. It mostly leads to high-output heart failure, but some cases of low-output heart failure have been reported, especially in patients with co-existing dilated cardiomyopathy [6,15,27]. One mechanism of high-output heart failure in thyroid excess is due to volume overload secondary to increased sodium, fluid retention, and activation of the renin-angiotensin-aldosterone (RAA) system [6,27,28]. Stimulation of erythropoietin production secondary to RAA activation and increased red blood cell (RBC) count adds to volume overload [27-29]. Heart failure in thyroid excess is associated with sinus tachycardia leading to an increase in cytosolic calcium in diastole and a subsequent decline in ventricular inotropy, diastolic dysfunction, and tachycardiomyopathy [8]. Isolated right-sided heart failure can be associated with tricuspid regurgitation and is due to pulmonary hypertension in thyrotoxicosis patients [8,28-30]. A rise in messenger ribonucleic acid (mRNA) levels that enhance the synthesis of contractile elements and sarcoplasmic reticulum Ca+ ATPase is the effect of thyroid hormones leading to calcium imbalance, especially in the heart muscle [29-32]. T3 has various effects on the heart at a molecular level. It affects the transcription of myosin chains in the heart and has a direct vasodilatory effect [5].

There is a relation between thyrotoxicosis and tachycardia-induced cardiomyopathy (tachy-cardiomyopathy or rate-related cardiomyopathy), wherein no other source of heart failure is found [32-34]. Thyrotoxic cardiomyopathy associated with heart failure is a dilated cardiomyopathy due to long-standing tachycardia and is reversible with treatment. Beneficial treatment includes anti-thyroid drugs, digoxin, beta-blockers, diuretics, and anticoagulation [30,32-35]. Because coexisting dilated cardiomyopathy can occur in Grave’s disease, it indicates the need for proper thyroid evaluation in a spectrum of cardiac disorders [28,34-38].

In any case of heart failure of unknown origin, thyroid excess is an important differential [32-34,37-39]. Even if the ECG shows normal sinus rhythm, heart failure in such cases should raise suspicion for thyrotoxicosis [35,37-39]. Elderly patients without any pre-existing cardiac conditions may present with apathetic or subclinical hyperthyroid states, wherein the measurement of thyrotropin is indicated. Restoration of normal cardiac function is usually seen in such cases with therapy against thyrotoxicosis [33,38-41].

Thyrotoxicosis and myocardial ischemia with ECG changes

Thyrotoxicosis can be attributed to overt changes in the ECG, such as intermittent ST-segment elevation [8,33]. Acute myocardial ischemia with transient ST segment elevation can be a consequence of iatrogenic hyperthyroidism in patients with normal coronary arteries without any occlusion [34]. There is an association between thyrotoxicosis and coronary artery spasm, leading to transient myocardial ischemia and anginal chest pain [35-37]. Moreover, T-wave inversion can be confused with effort angina in patients with thyrotoxic cardiomyopathy, and it might delay the identification of thyrotoxicosis as the causative etiology of cardiomyopathy and further worsen the prognosis [36-38]. Elevated free T4 (fT4) levels in the higher normal range might predispose to an increased risk of coronary artery disease in normal patients [37,39-41].

Thyrotoxicosis and amiodarone

Amiodarone is one of the most commonly used broad-spectrum antiarrhythmics, containing approximately 37% iodine by weight, and is one of the medications recommended in the advanced cardiac life support (ACLS) protocol [41-44]. It is a class III antiarrhythmic used to control various atrial and ventricular arrhythmias of the heart with simultaneous class I, II, and IV effects [45]. The effects of amiodarone on the thyroid are attributed to its high iodine content, direct toxic effects on the thyroid, and effects on thyroid hormone metabolism. Hypothyroidism presents more commonly than hyperthyroidism as a side effect of Amiodarone [41-45]. The thyrotoxic state can be sub-classified as type I amiodarone-induced thyrotoxicosis (AIT), usually associated with a pre-existing thyroid condition (e.g., nodular goiter in iodine-deficient areas), or type 2 AIT (drug-induced), presenting with a previously normal thyroid as thyroiditis or inflammation by interleukin-6 stimulation. Mixed AIT has features of hypo- and hyperthyroidism [40,45-48]. Treatment of type 2 AIT involves the use of prednisone at a dose of 30-40mg per day [44-48].

Amiodarone causes various changes in thyroid hormone metabolism. By inhibiting the 5’-deiodinase, it increases the serum levels of fT4, rT3, and TSH with a simultaneous decrease in fT3, and this counter-balance between fT4 and fT3 usually keeps the thyroid in a normal state. The blocking of peripheral deiodination in such a manner by amiodarone is responsible for its action on the heart. The decline of fT3 levels is responsible for most of the atrial and ventricular anti-arrhythmic effects of Amiodarone [41-44,48]. Strict monitoring of thyroid function is recommended while administering Amiodarone therapy [48-50].

## Conclusions

Atrial fibrillation can be the only manifesting symptom of thyrotoxicosis. Likewise, many other cardiac disorders might also be associated with thyroid dysfunction. There is a need to have a broad view of thyrotoxicosis and cardiac disorders. It is necessary to have a low threshold for suspecting thyrotoxicosis in heart failure, tachycardiomyopathy, and atrial fibrillation. Thyrotoxicosis has myriad effects on the heart, which are mediated by elevated levels of T3 interacting with intranuclear thyroid hormone receptors in the myocardium. A hyperthyroid state may lead to varied presentations, including exercise intolerance. It is important to evaluate thyroid function in patients with the presentations discussed above. Cardiothyrotoxic disease is common with advancing age. Arrhythmia is the most common presentation of cardiothyrotoxic disease. Significant complications, including heart failure, may arise in thyrotoxic patients, even in young individuals.

Treatment for the thyrotoxic cardiac disease should be directed towards heart rate control and the management of acute cardiovascular complications, and therapy directed to restore a euthyroid state can help attain improved cardiovascular outcomes. Treatment of underlying thyrotoxicosis should aim to correct the underlying cardiovascular manifestations. A thyrotoxic state leads to an elevated heart rate, systolic blood pressure, increased myocardial oxygen consumption with increased cardiac output, may induce angina symptoms, lower diastolic blood pressure, and systemic vascular resistance, all of which can be improved with thyroid-specific therapy to achieve a euthyroid state. It is important to recognize the impact of thyrotoxicosis on cardiac function, given the fact that achieving a euthyroid state may lead to a reversal of abnormal cardiac function.
